# Development of a core outcome set for use in community-based bipolar trials—A qualitative study and modified Delphi

**DOI:** 10.1371/journal.pone.0240518

**Published:** 2020-10-28

**Authors:** Ameeta Retzer, Ruth Sayers, Vanessa Pinfold, John Gibson, Thomas Keeley, Gemma Taylor, Humera Plappert, Bliss Gibbons, Peter Huxley, Jonathan Mathers, Maximillian Birchwood, Melanie Calvert

**Affiliations:** 1 Centre for Patient Reported Outcomes Research (CPROR), Institute of Applied Health Research, and Birmingham Health Partners Centre for Regulatory Science and Innovation, University of Birmingham, Birmingham, United Kingdom; 2 The McPin Foundation, London, United Kingdom; 3 Institute for Mental Health, School of Psychology, University of Birmingham, Birmingham, United Kingdom; 4 GlaxoSmithKline (formerly of CPROR, University of Birmingham), London, United Kingdom; 5 Addiction and Mental Health Group (AIM), Department of Psychology, University of Bath, Bath, United Kingdom; 6 Coventry and Warwickshire Partnership NHS Trust and Warwick Medical School, University of Warwick, Warwick, United Kingdom; 7 Centre for Mental Health and Society, Bangor University, Bangor, United Kingdom; 8 Institute of Applied Health Research, University of Birmingham, Birmingham, United Kingdom; 9 Mental Health and Wellbeing, Warwick Medical School, University of Warwick, Coventry, United Kingdom; 10 School of Psychology, University of Birmingham, Birmingham, United Kingdom; 11 NIHR Birmingham Biomedical Research Centre, NIHR Surgical Reconstruction and Microbiology Research Centre and NIHR Applied Research Collaboration West Midlands, University Hospitals Birmingham NHS Foundation Trust and University of Birmingham, Birmingham, United Kingdom; University of Auckland, NEW ZEALAND

## Abstract

**Background:**

A core outcome set (COS) is a standardised collection of outcomes to be collected and reported in all trials within a research area. A COS can reduce reporting bias and facilitate evidence synthesis. This is currently unavailable for use in community-based bipolar trials. This research aimed to develop such a COS, with input from a full range of stakeholders.

**Methods:**

A co-production approach was used throughout. A longlist of outcomes was derived from focus groups with people with a bipolar diagnosis and carers, interviews with healthcare professionals and a rapid review of outcomes listed in bipolar trials on the Cochrane database. An expert panel with personal and/or professional experience of bipolar participated in a modified Delphi process and the COS was finalised at a consensus meeting.

**Results:**

Fifty participants rated the importance of each outcome. Sixty-six outcomes were included in Round 1 of the questionnaire; 13 outcomes were added by Round 1 participants and were rated in Round 2. Seventy-six percent of participants (n = 38) returned to Round 2 and 60 outcomes, including 4 outcomes added by participants in Round 1, received a rating of 7–9 by >70% and 1–3 by <25% of the sample. Fourteen participants finalised a COS containing 11 outcomes at the consensus meeting: personal recovery; connectedness; clinical recovery of bipolar symptoms; mental health and wellbeing; physical health; self-monitoring and management; medication effects; quality of life; service outcomes; experience of care; and use of coercion.

**Conclusions:**

This COS is recommended for use in community-based bipolar trials to ensure stakeholder-relevant outcomes, facilitate data synthesis, and transparent reporting. The COS includes guidance notes for each outcome to allow the identification of suitable measurement instruments. Further validation is recommended for use with a wide range of communities and to achieve standardised measurement.

## Introduction

This article describes the development of a core outcome set recommended for use in community-based trials for adults with bipolar, as part of the PARTNERS2 study. The PARTNERS2 study aims to help integrate primary care and community-based mental health services, further methodological detail and rationale for methodological decisions are available in the published protocol [1]. The term “bipolar” is used throughout this paper and in this research in place of “bipolar affective disorder” [2], as the preferred term of research team members with lived experience of bipolar and Bipolar UK, the leading charity in this area. This been understood in this research in accordance with the definition and scope defined in the International Classification for Diseases (ICD-10) [2] (the ICD-11 will come into effect in 2022 [3]).

Bipolar is defined as “two or more episodes in which the [person]'s mood and activity levels are significantly disturbed, this disturbance consisting on some occasions of an elevation of mood and increased energy and activity (hypomania or mania) and on others of a lowering of mood and decreased energy and activity (depression)”. Life expectancy among people with a diagnosis of bipolar is reduced when compared with the general population [4–6] and the mortality gap appears to be widening [7]. In addition, individuals with bipolar experience increased unemployment and stigma [8], and bipolar research is under-researched when compared to other mental health research [9]. There is a growing primary evidence base that indicates the efficacy of psychological treatments (e.g. non-pharmaceutical interventions such as cognitive-behavioural therapy, family interventions, and psycho-education) for bipolar and its long-term management; however, meta-analyses are undermined by poor quality evidence [10]. A better understanding of interventions and to make comparisons between them requires data from well-designed and conducted randomised controlled trials (RCTs) [11] using a unified approach to outcome selection.

Trialists evaluate the effectiveness of an intervention in a clinical trial by choosing outcomes that reflect any beneficial or harmful effects—these can be specific, such as change in weight, or broad constructs, such as pain [12]. Outcomes can be measured in several ways, including the use of laboratory findings, biomarkers, or mortality; or they can be reported by observers, clinicians, or the patient themselves. RCTs can provide robust evidence to inform clinical decision making and health care policy development [13], but the inconsistent use of highly varied trial outcomes within the same research area can undermine evidence synthesis. Additionally, for outcome data to be useful, the outcomes used must be of relevance to a range of stakeholders [14], including people with a bipolar diagnosis, carers and healthcare professionals [12, 15]. Commonly, the outcomes used in bipolar research have focused on clinical outcomes, such as change in symptoms as assessed by clinicians. However a mounting view in mental health research suggests that a broader set of outcomes may better suit the goals that people with diagnoses seek to achieve during treatment. This has been indicated in the case of schizophrenia [16, 17], and the Bipolar Priority Setting partnership suggests this is also the case for those with a bipolar diagnosis [18].

A core outcome set (COS) is a standardised collection of outcomes recommended to be reported in all controlled trials within a research area [12]. A COS represents the minimum outcomes to be measured and reported when undertaking a trial [19]. A COS for use in trials for those receiving non-pharmaceutical community-based interventions for bipolar (rather than as a hospital in-patient) could reduce reporting bias and enable evidence synthesis. The aim of this research is to develop such a COS.

This is the first study to develop a COS for community-based bipolar trials but builds upon an effort in the field to unify the outcomes and priorities within bipolar research. In 2010, the development of two “core sets” for bipolar based on the International Classification of Function, Disability and Health (ICF) guide [20] began. Focusing on functioning, the core sets use the ICF guide for bipolar, and while they may be used as outcome measures in research settings, the main intention of these core sets are for use in clinical practice. In 2015 a set of “patient important outcomes” [21] were published, aiming to investigate the relative importance of bipolar outcomes from the perspective of patients. The set of “patient important outcomes” considered the views of a single stakeholder group, those of people with a bipolar diagnosis, whereas the COS developed in our research included a range of information sources and engaged the views of different key stakeholders, allowing for the potential identification of gaps [22]. In comparison with the COS developed in our research, the ICF study constructed a shortlist of treatment outcomes relevant in the evaluation and selection of pharmacological treatments and was not conceptualised with intention for use in community-based bipolar trials. Similarly, the ICF core sets [23] were not developed with the sole intention of its use to be in community-based bipolar trials, as has the COS described here. In 2016, the James Lind Alliance (JLA) published a Priority Setting Partnership (PSP) about bipolar [24]. This provides researchers with 10 new bipolar research priorities into which the COS could be adopted and demonstrates increasing interest in bipolar research. The Royal College of Psychiatrists have also provided an overview of outcome measures for use in adult psychiatry [25].

## Methods

### Ethics statement

Ethical approval was sought and granted from the National Research Ethics Service (NRES) West Midlands—Edgbaston (Reference Number: 14/WM/0052). The research was registered with the COMET (Core Outcome Measures in Effectiveness Trials) Initiative [26] and is reported in adherence with the COS-STAR Statement [27] and the GRIPP2-SF [28]. The study was conceived prior to publication of the COS Standards for Development [29] recommendations but is in alignment with its standards.

A co-production approach, drawing upon the expertise of academics, healthcare professionals, people with bipolar, and carers, was using in this research. Experiential expertise from people with a bipolar diagnosis and carers was facilitated through the PARTNERS2 patient and public involvement (PPI) programme. Peer researchers with a diagnosis of bipolar were employed within the research team and three Lived Experience Advisory Panels (LEAPs) were established, consisting of an average of 5 people with schizophrenia and bipolar diagnoses and family members with experience of caring for individuals with mental health diagnoses. Research team members and advisors with lived experience advised on and provided strategies to ensure the research phases and output would be relevant and useful to those with bipolar. Research team members with lived experience were involved in all of the tasks required for the fulfilment of the research, including collection and analysis of data, in the same manner as those without lived experience. However, research team members with lived experience had an additional role whereby they would provide ongoing advice to colleagues that would ensure the research and the manner in which it was conducted remained relevant and appropriate to people with bipolar, for whom the COS would be finally intended. In this paper, references to the “research team’, this should always be taken to include those research team members with lived experience. The LEAPs discussed and advised on the phases of COS development on 8 occasions between February 2015 and November 2016. The LEAPs often discussed the same phases at multiple meetings. Work undertaken by LEAP members included commenting and advising on consent and information materials (in particular ensuring use of accessible language), recruitment strategies, and delivery of research. Meetings were held with individual members of the LEAP on two occasions to pilot the Delphi interface.

**Fig 1:**
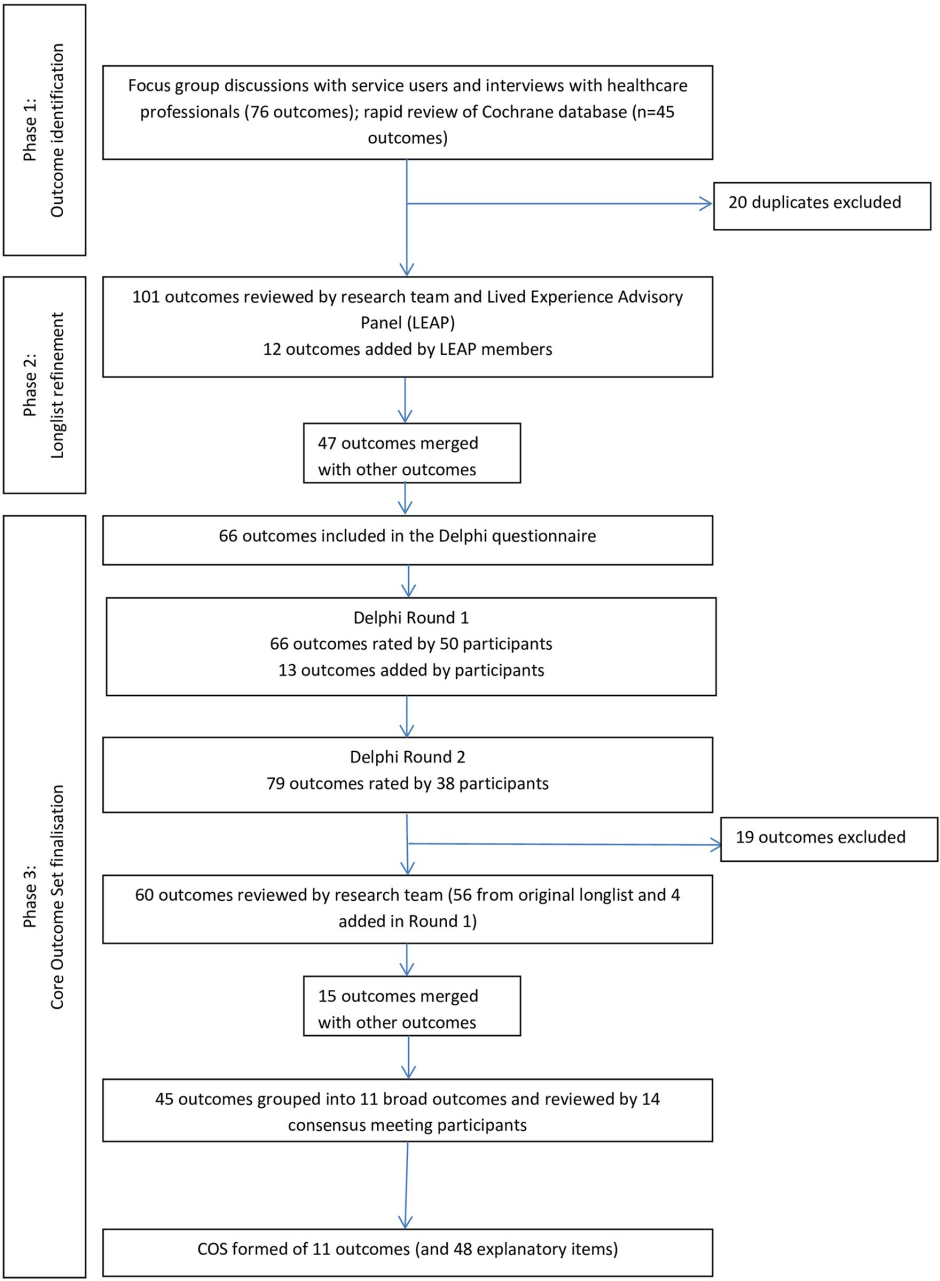
Illustration of core outcome set development process.

**Fig 2:**
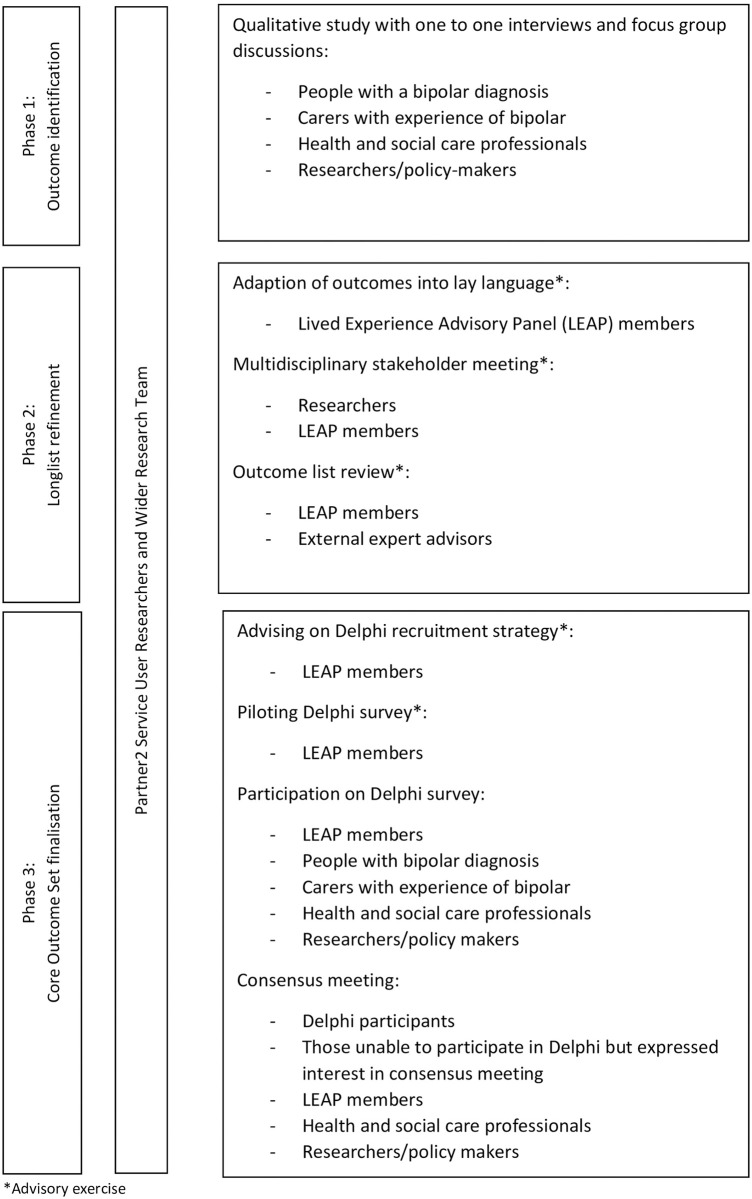
Overview of stakeholder involvement per phase.

This article details the process through which an outcome longlist was developed and subjected to rating and refining via a two round Delphi survey and a stakeholder consensus meeting, resulting in a COS recommended for use in community-based bipolar trials. The COS was developed via three phases: 1) identifying a longlist of outcomes from focus group discussions, one-to-one interviews, and a rapid review, 2) refining the outcome longlist using Delphi methodology, and 3) finalising the COS in a consensus meeting as the last part of the Delphi (see Fig 1). The three phases involved input from several stakeholders (see Fig 2).

The authors can confirm that the development of the bipolar COS reported was undertaken in adherence to the published protocol (steps 1–3), with the exception of use of 2 rounds of Delphi survey instead of 3, to mediate sample attrition. The latter steps of the published protocol, 4 and 5, will be completed and reported separately. The schizophrenia COS is still in development.

### Phase 1: Outcome identification

Outcomes were identified through a qualitative study involving focus group discussions with people with a bipolar diagnosis and carers with experience of bipolar; one-to-one interviews with healthcare professionals and researchers; and a rapid review of the Cochrane database.

#### Qualitative sampling, recruitment and data collection

People with a bipolar diagnosis and carers were recruited in the West Midlands and Lancashire regions of the UK through local support groups, electronic advertisement via third sector organisations, and snowball sampling. Eligible participants self-identified as having a bipolar diagnosis (current or previous); receiving/having received mental health treatment in a community setting; being aged between 18–65 years, as recommended by clinical research team members; and fluent in English. Eligible carers self-identified as having supported individuals with bipolar diagnoses as a family carer; being aged between 18–65 years; and fluent in English. Consent was taken at entry to the study and re-taken at the focus group. Focus group discussions were co-facilitated by two research team members, one of whom is a “peer researcher” with experience of mental health problems in addition to having a research background. This approach is intended to build rapport and trust with participants [30] through self-disclosure. Participants were asked open questions [31] (see S1 File) and produced written descriptions of the effects of their mental health problems on daily living which were placed on the walls of the room, allowing their categorisation under broad headings. This exercise of “concept mapping” [32] facilitated discussion and engagement from all participants, and informed subsequent analyses.

Researchers and healthcare professionals were recruited through existing professional contacts of the research team, aided by the prominent members of the bipolar research field being known to the team. Purposive sampling was used to capture a range of professional roles including health care professionals, social care professionals, commissioners, researchers and policy makers (see S2 File). Eligible participants were professionally involved with people with bipolar diagnoses. Semi-structured telephone interviews were undertaken by TK (see S1 File). Verbal consent was taken at the start, within the digital audio recording of the interview, and participants returned completed consent forms to the researcher after the interview.

#### Analysis of focus group discussions and one-to-one interviews

Focus group and interview recordings were transcribed verbatim and checked for accuracy by TK through concordant listening and reading. Data from focus group discussions and one-to-one interviews were analysed together due to the purpose of analysis being to identify all possible outcomes. Transcripts were uploaded to Dedoose online qualitative data management software [33] to manage and support data analysis. Dedoose was used to organise the qualitative data collected during the focus groups and one-to-one interviews to generate the outcome longlist. Descriptive accounts of the interviews and focus group discussions were written (TK), focusing on outcome identification. The first iteration of the coding structure was developed following thorough reading of the early transcripts and written descriptions from the focus group discussions (TK). These were open coded, line by line, and these codes were grouped into categories. Over-arching themes were identified from the categories to generate detailed outcome lists. Code formation was completed collaboratively by peer researchers and other research team members (VP, RS), drawing on personal experiences and prompting reflexive discussion and detailed conversations within the wider team. A 20 percent code application check was completed (RS).

#### Rapid review

A pragmatic approach was used to identify outcomes collected in bipolar research in community settings. Two researchers (TK, GT) independently performed a complete search of all pre-categorised titles listed under the bipolar reviews on the Cochrane database for systematic reviews. Cochrane reviews follow a rigorous structure and outcomes listed under the “Primary Outcomes and Secondary Outcomes” sub-headings in each review’s methods section were identified. Data relating to measures used and monitoring adverse effects or safety (unless these were included in the primary and/or secondary outcomes listed) were not extracted. Review protocols were not included in this data extraction. The lists of outcomes formed by each researcher were compared for completeness and differences in the categorisation were resolved through discussion (Database accessed in March 2015).

### Phase 2: Longlist refinement

The outcomes identified in Stage 1 were checked for duplication though detail was favoured at this stage. With the input of the LEAPs, the outcomes were adapted into lay language, organised under broad headings, and merged to minimise overlap. The outcome list was then reviewed during a multi-disciplinary stakeholder meeting composed of four mental health researchers including two with personal experience of bipolar, three outcome measurement researchers, and LEAP members including three people with a bipolar diagnosis and a carer. The resulting outcome list was then reviewed by the wider PARTNERS2 research team, LEAP members and external expert advisors to consider the merging decisions to ensure the list was comprehensive to the best of their knowledge.

### Phase 3: Core outcome set finalisation

The outcome longlist developed during Stages 1 and 2 were subjected to a two-round Delphi survey and a final consensus meeting.

#### Delphi survey

Participants were recruited from the UK only. People with a bipolar diagnosis and carers were recruited nationally through local support groups, electronic advertisement via third sector organisations and social media. The LEAPs advised on recruitment strategies and circulated recruitment materials via their own networks. Health and social care professionals and researchers were recruited through the professional networks of the PARTNERS2 research team. Purposive sampling was used to capture a range of professional roles and supplemented as required through snowball sampling.

The main eligibility criteria were that participants had experience of bipolar, due to receiving a diagnosis themselves, caring for someone who had a diagnosis, working in a professional capacity with those with bipolar, and/or a research background in bipolar, and that they could take part in both rounds of the Delphi. Following advice from the research team members with lived experience during the course of the research, a screening tool was also used to promote representativeness in the Delphi sample so that it would be more typical of the diverse population of those with bipolar (see S2 File). With their input, the screening tool was developed with particular focus on diversity of age, ethnicity, gender, and history of mental health support for participants with lived experience of bipolar. This was used to monitor sample diversity and inform and direct recruitment. To promote inclusion, a paper-based version of the survey was available upon request.

The Delphi survey was hosted by Delphi Manager software [34] and piloted with two LEAP members using cognitive appraisal techniques [35] (AR), resulting in changes to wording and providing insight into how questions were interpreted. The length of time taken to complete each round of the survey was noted to be 30 minutes, and this was included in consent information provided to potential participants. The survey design presented participants with an outcome label and an option to read a description of the outcome. These descriptions were generated by the LEAPs and meant participants could choose how much information they needed to read (see S3 File). During development of the outcome list, the outcomes were presented in domains: recovery, connectedness, mental health, physical health, self-management, medication, quality of life, service outcomes. These were developed by the research team, including research team members with lived experience, and were approved by the LEAPs. The purpose of the domains was to organise the outcomes and to promote accessibility during development. It was decided by the research team and LEAP advisors that the outcomes should be presented in these domains in the survey, for the same purpose.

During Delphi registration, participants assigned themselves to one of four stakeholder groups, 1) person with a bipolar diagnosis, 2) carer, 3) health/social care professional, 4) researcher/policy maker. Participants were requested to choose the group to which they most identified, though it was acknowledged that they may belong to more than one. They were invited to rate each of the outcomes on the longlist on a nine-point Likert scale (where nine indicated the highest level of importance and one indicated the lowest). Participants were also invited to suggest outcomes they considered were absent from the Stage 1 and 2 longlist. These were automatically included, verbatim, for rating in the Round 2. Following closure of Round 1, the software internally calculated the ratings of each outcome by stakeholder group. Participants returning for Round 2 were presented percentage distribution of scoring for each point on the scale from 1–9 from the previous round, along with their own scores for each outcome. Round 2 participants were invited to review their own ratings from the first round and consider whether they wished to change their initial score for each outcome, using the same scale. All original outcomes presented in Round 1 were presented in Round 2. Further details pertaining to methodology can be found in the published protocol [1].

#### Analysis of Delphi survey data

The conditions and means for determining inclusion and exclusion were defined in advance [36]. For each outcome presented in Round 2, the proportion of participants rating 1–3, 4–6, and 7–9 on the Likert scale was calculated. Outcomes rated as 7–9 by >70% of participants and 1–3 by <25% of participants were pre-specified in our protocol to be automatically included in the COS. Outcomes automatically excluded from the COS were those which >70% of participants rated as 1–3 and <25% rated as 7–9. “Disagreement” occurred when >33% of participants scored an outcome as 1–3 and >33% scored the same outcome 7–9. These outcomes underwent additional analysis whereby their mean scores were calculated and those outcomes with a mean above 4.5 were included in the COS and those with a mean less than 4.5 were excluded. These criteria are comparable to those used throughout COS methodology [37–39].

#### Consensus meeting

Delphi participants were approached and invited to participate in the consensus meeting, as were those who had been unable to participate in the Delphi but had expressed an interest in attending the consensus meeting. LEAP members, members of the wider PARTNERS2 research team with professional experience as mental health professionals who had limited involvement in the COS development to date, and known contacts of the research team were also invited to participate. A screening tool was used to promote diversity (see S2 File).

The original aim of the consensus meeting was for attendees to discuss those outcomes that were in “disagreement”. Due to the large volume of outcomes that were rated as “important” by Delphi participants, the outcomes automatically included in the COS following the Delphi analysis were provisionally grouped by the research team using the domain headings in which they were presented during outcome list development and then in the Delphi survey. This is an adaption to the standard COS methodology. The proposed grouping of outcomes was finalised at a consensus meeting. Attendees voted on grouping of outcomes and rearranged them as they saw fit. Decisions made during the consensus meeting were subject to anonymous voting using TurningPoint software [40] and only those decisions sanctioned by >70% of the group attendees were ratified. However, in all cases where there was less than 100% consensus, the decisions were discussed further until those who were in disagreement were satisfied that their views had been considered and that the decision could proceed.

## Results

### Phase 1

Three focus group discussions were held, two with people with a bipolar diagnosis and one with carers. The groups ranged in size from 4–8 people and lasted between 96–120 minutes. Recruitment and data collection took place between July 2014 and March 2015. Fifteen people with a bipolar diagnosis with an average age of 46 years participated; 9 identified as female and 6 as male; 12 identified as White British and 3 identified as British Asian or Asian. Seven carers with an average age of 59 years participated; five of these identified as female and 2 as male; five identified as White British, 1 as British Asian or Asian, and 1 did not specify their ethnic background.

Telephone interviews were carried out with 16 healthcare professionals and researchers. Interview length ranged from 25–47 minutes. Recruitment and data collection took place between July and November 2014. Participants held multiple professional roles: 2 clinical commissioners, 2 non-clinical commissioners; 4 general practitioners; 4 healthcare management/mental health leads; 1 mental health nurse; 5 psychiatrists; 6 researchers; 2 social workers; and 2 third sector employees.

Data were extracted from 17 bipolar reviews contained within the Cochrane database and 45 independent outcomes were identified from the bipolar database. Outcomes were classed as “independent” if the terminology used in the Cochrane database showed a clear difference. If the outcome terminology showed notable similarity, such as mortality and mortality rates, this was classed as one independent outcome. The majority of these outcomes were used in multiple reviews. Seventy-six outcomes were identified through the focus group discussions and interviews. Twenty of these were removed due to duplication.

### Phase 2

One hundred and one outcomes were reviewed by the research team and LEAPs, resulting in the addition of 12 outcomes and the merging of 47 (see S4 File).

### Phase 3

Fifty Delphi participants were recruited. Delphi participants were recruited between September and December 2016, during which Round 1 was open. All participants participated via the online survey. Round 2 was open from December 2016-February 2017. Ninety-three individuals were contacted via known and referred contacts of the research team and a 32% (n = 30) recruitment rate was achieved. Twenty participants were recruited through the LEAPs, support groups, social media, and third party organisations.

A process of monitoring and reminders was used to ensure completion, resulting in a 76% return rate (n = 38) to Round 2 of the Delphi (see S5 File). Fifteen people with a bipolar diagnosis participated, 2 of whom identified as male and 13 as female; 12 identified as White British, 1 as British Asian or Asian, 1 as mixed heritage, and 1 did not specify. While the screening tool was used to promote sample representativeness, nobody was excluded from the Delphi on this basis (see S6 File). Four carers participated, 1 identified as male and 3 as female; 3 identified as White British and 1 as British Asian or Asian. Twenty-three healthcare professionals participated, 12 identified as male and 11 as female; 19 identified as White British, 2 as British Asian or Asian, and 2 did not specify. Eight researchers participated, all identifying as White British, 1 identifying as male and 7 as female.

Sixty-six outcomes were included in the Delphi questionnaire and 13 outcomes were added by participants during Round 1. Three outcomes were suggested by Round 1 Delphi participants and rated as important by participants in Round 2, so were included in the consensus meeting discussion. A total of 60 outcomes met the pre-specified criteria for automatic inclusion into the COS (see S7 File), 56 from original longlist and 4 added in Round 1 by Delphi participants. Fifteen of these outcomes were merged with other outcomes by the research group and then the remaining 45 outcomes were provisionally grouped into 11 outcome domains, (see S8 File) to ensure the final COS would be feasible for use in future trials.

The consensus meeting, co-chaired by an outcome measurement researcher and a peer researcher, took place in September 2017 and was attended by 14 people: 6 healthcare professionals, 5 people with a bipolar diagnosis, 2 carers and 1 researcher. Table 1 shows the results of the voting and discussion.

**Table 1:** Final core outcome set, voting rounds and scores.

Outcome and explanatory items	Total number of voting rounds	Final voting results		
		Keep	Change	Discuss further
**Personal Recovery**	2	93%	7%	0%
Achieving goals; having a sense of identity; hope; meaning in life; empowerment; coping with self-stigma; wellbeing; self-esteem (which may overlap with “mental health and wellbeing”); and being able to build an everyday life				
**Connectedness**	1	79%	0%	21%
Satisfaction with social networks; trust; relationships with friends, family and others; social support via a person’s own social contacts; social isolation; and loneliness (“loneliness” was considered an important concept that could be an outcome itself or could overlap with “mental health and wellbeing”)				
**Clinical recovery of bipolar symptoms**	1	100%	0%	0%
A person’s increased or reduced experience of paranoia; delusions; anxiety; depression; unusual behaviour; elevated mood; and a person’s relapse or recovery response				
**Mental health and wellbeing**	1	79%	7%	14%
A person’s experience of psychological distress; and guilt and shame				
**Physical health**	1	100%	0%	0%
Relates particularly to the health concerns for people with bipolar including cardiovascular disease, metabolism concerns, or substance use but the focus of this will differ from trial to trial				
**Self-monitoring and management**	1	100%	0%	0%
Self-management and understanding of diagnosis; self-management of medication; medication adherence underpinned by satisfaction with medication; mood control and stabilisation; increasing healthy behaviours and reducing unhealthy behaviours insofar as they are linked to their impact upon bipolar				
**Medication effects**	3	79%	21%	0%
Side-effects; coping with side-effects; and satisfaction with medication				
**Quality of life**	1	93%	7%	0%
Health-related quality of life; meaningful occupation and activities; being in control of finances; personal safety and security; home living conditions and organisation; and vulnerability to harm				
**Service outcomes**	1	86%	7%	7%
There being a relapse plan in place; timely and accurate diagnosis; and number of days between referral and subsequent assessments				
**Experience of care**	1	100%	0%	0%
Dignity and respect; a person’s overall satisfaction with service; shared decision-making and control; a trusting patient and healthcare professional relationship; and active involvement of the person in their in treatment and care plan				
**Use of coercion**	1	93%	7%	0%
The use of measures such as sectioning, restraint, isolation, or seclusion to manage distress during hospital admission				

### Final core outcome set

The outcome identification, refining and finalisation are illustrated in Fig 1. The final COS included 11 outcome domains. The adapted process meant moving from detailed outcome items in the Delphi, to grouped outcomes in the consensus workshop.

The consensus meeting participants finalised the COS and mapped individual long list items to each outcome as follows, providing a guide for future use (Table 1).

Recommended safety indicators were: all-cause mortality, self-harm, attempted suicide, self-harm, use of emergency care and recommended outcomes for health economics evaluation included all health service use including hospital admission, home treatment, and outpatient use. All (n = 14, 100%) consensus meeting attendees voted in favour of these (see S9 File).

## Discussion

The final COS consists of 11 outcome domains: personal recovery; connectedness; clinical recovery of bipolar symptoms; mental health and wellbeing; physical health; self-monitoring and management; medication effects; service outcomes; service user experience of care; and use of coercion. The development of the COS has drawn upon several sources, including a rapid review of the Cochrane database and qualitative work with key stakeholders. We recommend that researchers use this COS to inform their selection of measures used in future community-based bipolar trials. There are a range of measures currently available for use, however, we would recommend that when choosing measures, teams do so with consultation from key stakeholders including methodologists, clinicians, and those with lived experience, in addition to considering psychometric properties of measures and their alignment with research aims. Further research is required to identify which measures should be recommended for each COS outcome, however these would require regular review and may vary due to the particular requirements of each study.

The longlist of 66 outcomes first included in the Delphi survey were rated highly by participants, and as a result, 56 (85%) of these were automatically included in the proposed COS arrangement discussed at the consensus meeting. This suggests the process of outcome identification and refining used in this work has generated a large number of outcomes that were relevant to the stakeholders involved. Bipolar is a condition that impacts on every aspect of a person’s life, and thus the detailed outcome identification undertaken within the COS process reflected this extensive process and all-encompassing impact. However, use of the COS would need to be feasible in trials while retaining all outcomes rated as important by participants. Grouping items into higher-level outcome domains during the consensus meeting served this purpose. The large number of items included in the final COS was discussed at length amongst the research team and at the consensus meeting. It was felt it was important to adhere to our protocol and retain items that met the pre-specified threshold, particularly as stakeholders had strongly indicated their importance. There is potential for several items to be assessed together, for example, using health related quality of life or satisfaction questionnaires. Core outcomes sets recommend ‘what’ to measure; further research is required to evaluate the measurement properties of assessment tools, map these to the outcomes identified and reach consensus on the optimal ways to assess these outcomes in a standardised way.

Ongoing validation is necessary to ensure external validity; the continued relevance and importance of the outcomes; to evaluate implementation; and engage additional stakeholders [1]. This research was undertaken with inclusion of participants based in England, Wales and Scotland. Further research is required to assess the validity of the COS in specific populations, such as black, Asian and minority ethnic (BAME) communities in the UK, and to achieve greater consensus on its applicability in international settings, involving expert panels and stakeholders from the widest possible range of nations and communities. Efforts are also required to ensure the adoption and endorsement of the COS with funders, journals, and others involved in the development, facilitation and undertaking of bipolar research. Uptake of the COS [41], its implementation [42], and the consistency of its measurement [43] can be assessed through review of future community-based bipolar trials and their publication outputs.

The strengths of this research include that stakeholders were given multiple opportunities throughout to identify and remedy any gaps in the outcomes longlist and proposed core outcome set as well as the consensus generating nature of the Delphi. The relevance and importance of the COS is greatly strengthened overall through extensive lived experience input at each stage. In addition to the contribution of people with a bipolar diagnosis and carers as research participants in the qualitative component, this work has drawn upon the expertise of peer researchers employed on PARTNERS2 and LEAPs throughout the process, including in the analysis of results. This ensures the COS has validity for people directly experiencing bipolar and seeking support and treatment, as well as for health care professionals, researchers and commissioners. Of the researchers gathering initial qualitative data, one had personal experience of bipolar; three LEAPs were consulted regularly and redefined and reworded the outcome descriptors for the Delphi survey; the Delphi and consensus meeting included equal numbers of professionals and people with lived experience of bipolar; and the consensus meeting was co-chaired by a peer-researcher with a bipolar diagnosis. Deliberations involving this full range of stakeholders on equal terms led to a more accessible and salient COS. Each outcome included in the COS is accompanied by an explanatory guide to aid interpretation and facilitate the selection of suitable measures without ambiguity about the intended meaning as understood by our participants, team members and advisors. The resulting COS aligns with accepted definitions of outcomes, indicated notably in its inclusion of outcomes relating patient experience [25].

Limitations include that the rapid Cochrane review identified outcomes used in systematic reviews which had the benefit of allowing a rapid and practical review, however the elicitation of outcomes through this methodology may not be complete as systematic reviews will not include all outcomes used in research within a given field. In addition to this, the outcomes identified in this way may not have been chosen by a range of stakeholders including those with lived experience, clinicians, and researchers.

Further limitations relate to the Delphi sample. The first is that the final sample 50 is relatively small, particularly given that four stakeholder groups were recruited. Secondly, is the issue of participant attrition between rounds during the Delphi survey. This is, however, mediated because the data collected in the first round was included in the summarised results presented to Round 2 participants regardless of whether the corresponding participant had returned, and would have been used to inform the Round 2 ratings. Additionally, Delphi participants were asked to self-assign to one of four stakeholder groups during survey registration– 1) person with a bipolar diagnosis; 2) carer; 3) health/social care professional; 4) researcher/policy maker. During the Delphi development it was indicated that participants may identify with more than one of these categories and as such, they were invited to assign to whichever group with which they identified primarily. Open text allowed participants to further elaborate about the breadth of their experience, however these were not able to be included in analyses. Further to this, while efforts were made to ensure the diversity of the sample of individuals recruited, men and those from BAME communities, specifically those of Black, African, Caribbean, Black British origin, are underrepresented. This may be addressed with additional validation exercises with further groups and communities.

This research has used robust methodology to develop a COS for community-based bipolar trials. Its adoption in future studies will enable the generation of coherent, stakeholder-relevant outcome data that may strengthen meta-analyses and promote the value of bipolar research and the integration of subsequent findings into clinical practice.

## Supporting information

S1 File (DOCX)

S2 File (DOCX)

S3 File (DOCX)

S4 File (XLSX)

S5 File (DOCX)

S6 File (DOCX)

S7 File (DOCX)

S8 File (DOCX)

S9 File (DOCX)
