# Supplementary material for: Development of a core outcome set for use in community-based bipolar trials—A qualitative study and modified Delphi
Source: PLoS One. 2020 Oct 28;15(10):e0240518. doi: 10.1371/journal.pone.0240518 (PMC7592842; doi:10.1371/journal.pone.0240518)
Supplement: S1 File — (DOCX) [file pone.0240518.s001.docx]

**PARTNERS2 – Service user focus group topic guide**

1. How have your mental health problems changed your life and what have you lost* because of them?
2. What are your goals in living with your symptoms?
3. What’s important to you about your health and about your life?
4. Is there anything that you feel you’re not able to do now that maybe you used to be able to do?
5. When you were first diagnosed, did you have different goals or expectations? If so, how have your goals changed?
6. Thinking back over what we have discussed, do you think that these are the things researchers should be looking at? Are there things which are not suitable to measure?

**PARTNERS2 – Healthcare professional interview topic guide**

1. How does Bipolar affect a person’s life? What do they lose*?
2. What outcomes are you looking to achieve when delivering care or support to people with bipolar?
3. Do these outcomes vary between different people on your case load? And how?
4. Are different outcomes important to patients at different stages in their illness? At different stages in their health? Controlled vs Stable? Diagnosis vs later management?

* It was felt that the subject of “loss” was important to acknowledge and considering loss, though painful, can enable people to consider what they want to get from treatment – maybe something to mitigate or cope with loss.
