# Supplementary material for: Development of a core outcome set for use in community-based bipolar trials—A qualitative study and modified Delphi
Source: PLoS One. 2020 Oct 28;15(10):e0240518. doi: 10.1371/journal.pone.0240518 (PMC7592842; doi:10.1371/journal.pone.0240518)
Supplement: S2 File — (DOCX) [file pone.0240518.s002.docx]

**Researcher and healthcare professional participant targets:**

Psychiatrists (5); Team Managers (5); General practitioners with special interest (5); CCG mental health leads (5); Commissioners, including/ideally joint commissioners (5); Researchers (5); Health and well-being board MH representation (2); Director of public health (2)

**Screening format for potential participants:**

*Aim 1: recruit sample with a diagnosis of bipolar (25) or experience caring for someone with a diagnosis of bipolar (25) from a range of age groups, approximately 20% BME participants (5), and 50% gender split (~12 of each). For individuals with bipolar, 25% should be off medication (~6) and 75% should be on (~18); 50% of individuals should be receiving support from primary care (~12) and 50% (~12) should be receiving support from secondary care; range of length of diagnoses.*

*Aim 2: recruit healthcare professionals (25) and policy-makers (10) who have expertise relating to bipolar disorder. The policy-maker sample may also include researchers (5).*

*Purposive sampling will be used on a rolling basis and individuals will be recruited based on the answers provided during the screening process according to the diversity requirements of the sample to date.*

When contact has been made with a potential service user participant, ask the following questions:

1. **Do you have a bipolar diagnosis**? (if yes, proceed to question 4, if no, proceed to question 2)
2. Do you have experience of caring for someone with bipolar disorder? (if yes, proceed to question 4, if no, proceed to question 3)
3. **Are you a healthcare professional, policy-maker, or researcher with experience of working with individuals with a bipolar diagnosis, or have expertise relating to bipolar disorder?** (If yes, proceed to question 4. If no, end screening and thank them for their interest but explain how this research relates specifically to individuals who have a diagnosis of bipolar, or have experience caring for a person with a diagnosis of bipolar, or healthcare professionals, policy-makers, and researchers with experience/expertise relating to bipolar disorder.)
4. **Are you able to commit to a complete 2 questionnaire over the next three months? They take [X amount of time] and it is very important that, should you decide to take part, that you complete both rounds of the questionnaire. Do you feel this is something you would be able to do?** (If yes, proceed to question 5. If no, end screening and thank them for their interest but explain the importance of taking part in both rounds of the questionnaire and why the research team needs the information provided by completing both rounds of the questionnaire.)
5. **Do you feel comfortable completing the questionnaire using an online format?** (If yes, for service user participants, proceed to Diversity Screening section. If yes, for carers, researchers, health professionals, and policy-makers, request email information to send the information sheet or read aloud the information sheet for telephone consenting. Explain the subsequent stages of contact. If no, proceed to question 6.)
6. **If you were provided with a paper copy of the questionnaire, would you be happy to participate?** (If yes, for service user participants, proceed to Diversity Screening section. If yes, for carers, researchers, health professionals, and policy-makers, request postal information to send information sheet, consent form, and questionnaire. If no, proceed to question 7.)
7. **Would you be interested in completing the questionnaire over the phone?** (If yes, for service user participants, proceed to Diversity Screening section. If yes, for carers, researchers, health professionals, and policy-makers, make arrangements for either sending information sheet by post or reading information sheet over the phone, taking consent, and arranging telephone questionnaires. If no, thank them for their interest and explain that they will not be able to take part.)

**DIVERSITY SCREENING:**

*Complete this section with potential service user participants who have answered required questions to reach this point.*

| **Question** | **Characteristic** | **Yes** | **No** |
| --- | --- | --- | --- |
| 1. What is your ethnicity? | BME? |  |  |
| 1. Are you currently taking medication for your bipolar? | Medication? |  |  |
| 1. Are you currently receiving support from primary care? | 1° Care? |  |  |
| 1. Are you currently receiving support from secondary care? | 2° Care? |  |  |

1. What gender do you identify with?

| Male | Female | Other |
| --- | --- | --- |

1. What is your age (years)?

| 18-30 | 31-40 | 41-50 | 51-60 | 61-70 | 71+ |
| --- | --- | --- | --- | --- | --- |

1. How long have you had a bipolar diagnosis (years)?

| <2 | 3-10 | 11-20 | 21-30 | 31-40 | 41+ |
| --- | --- | --- | --- | --- | --- |

*Based on the answers to the above questions, compare information with the current pre-Delphi sample, if participant meets current requirements, proceed to requesting relevant information required for electronic, postal, or telephone questionnaire. If not, thank them for their interest but explain that the study is currently recruiting different groups and ask if it would be possible to get in touch at a later date if these requirements change. If yes, request relevant contact information and if no, thank them for their time and their interest in the research.*
