# Supplementary material for: Development of a core outcome set for use in community-based bipolar trials—A qualitative study and modified Delphi
Source: PLoS One. 2020 Oct 28;15(10):e0240518. doi: 10.1371/journal.pone.0240518 (PMC7592842; doi:10.1371/journal.pone.0240518)
Supplement: S3 File — (DOCX) [file pone.0240518.s003.docx]

| **1** | **1. PERSONAL RECOVERY** | **Description** |
| --- | --- | --- |
| 1.01 | Achieving goals | A person is able to set significant personal goals, and to make changes and realistic plans to work towards or achieve them. |
| 1.02 | Sense of identity | A person has confidence in own identity, including abilities and personal worth; trusts self to make decisions and take action; feels taken seriously, accepted and valued by others. |
| 1.03 | Hope | A person accepts that the future can be positive and is able to recognise and cope with feelings of hopelessness and despair. |
| 1.04 | Meaning in life | A person finds purpose and structure in life; able to participate in activities, roles or work that hold personal significance. |
| 1.05 | Empowerment | A person feels able to organise own life, make choices, determine own future, challenge assumptions and to live independently, recognising the importance of giving and receiving mutual support |
| 1.06 | Coping with self-stigma | A person is able to cope with own negative thoughts and feelings about mental illness or bipolar diagnosis, and their potential to damage confidence and limit expectations |
| 1.07 | Well-being | Feeling comfortable, healthy and happy including both physical and mental health |
| 1.08 | Self-esteem | A person’s confidence in their own worth and intrinsic value as a human being. Self-esteem may overlap with the “mental health” outcome. |
| 1.09 | Able to build an everyday life | A person is able to build an ordinary, everyday life, which may include education, work, housing, family, and financial security, and sustain this through episodes of illness. |
| **2** | **2. CONNECTEDNESS** |  |
| 2.01 | Satisfaction with social networks | A person is able to establish, maintain and, where needed, repair social connections, including meeting people, joining activities and being involved in communities. Despite being part of a “social network”, the person must be satisfied within theirs. |
| 2.02 | Trust | A person has confidence in relationships, establishes close, trusting and respectful relationships with others |
| 2.03 | Relationships with friends, family and others | A person can develop appropriate connections with family, friends and others and maintain these during episodic mental health crises |
| 2.04 | Social support | A person is able to get assistance from other people when needed, and is part of a mutually supportive social network. This refers to a person’s own social contacts rather than formal service provision (for example, housing, benefits, et cetera). |
| 2.05 | Social isolation | A person is alone and lacking close relationships, avoiding or failing to contact other people |
| 2.06 | Loneliness | A person is isolated and without close friends, company or support from others. This item is of such importance to stakeholders that it could stand alone or overlap with the “mental health” outcome. |
| **3** | **CLINICAL RECOVERY OF BIPOLAR SYMPTOMS** |  |
| 3.01 | Paranoia | Thoughts or delusions of persecution, preference to self in everyday experience. |
| 3.02 | Delusions | Holding strongly to a false belief, idea or impression despite evidence to the contrary |
| 3.03 | Anxiety | Feelings of constant worry, or deep concern or uneasy about uncertainties |
| 3.04 | Depression | Feelings of severe sadness and unhappiness, often with decreased energy, constant feelings of guilt, doubt or self-blame, worthlessness and hopelessness. |
| 3.05 | Unusual Behaviour | Behaving in ways that are out of character for the person and which lead to problems for the person and for other people. |
| 3.06 | Elevated mood | A time of excessive energy, little sleep, pressure of thoughts that can be grandiose or deluded; can involve loss of judgement, while lacking awareness. This replaces “manic state”. |
| 3.07 | Relapse or recovery response | Symptoms and ability to cope worsen, leading to increased need for support, may include use of crisis services or admission to hospital |
| **4** | **MENTAL HEALTH AND WELLBEING** |  |
| 4.01 | Psychological distress | Non-physical, psychological pain and/or distress |
| 4.02 | Guilt and shame | Feelings of excessive or unmanageable embarrassment, shame, guilt or remorse related to past behaviour, situations or relationships. |
| **5** | **PHYSICAL HEALTH** |  |
| 5.01 | Physical health | The physical health of the person, including disease, illness and physical functioning. This may include more detail, in particular the health concerns for people with bipolar including cardiovascular, metabolism, substance use etc. The focus of this will differ from trial to trial. |
| **6** | **SELF-MONITORING AND MANAGEMENT** |  |
| 6.01 | Self-management and understanding diagnosis | A person understands factors that maintain mental health or can trigger bipolar symptoms, including personal “relapse signature” such as triggers, early warning signs and strategies that help to manage own condition. |
| 6.02 | Self-management of medication | Able to manage medication in cooperation with health care professionals |
| 6.03 | Medication adherence | Taking medication that is prescribed. This relates to a person’s satisfaction with medication and their adherence as a result. |
| 6.04 | Mood control or stabilisation | A person accepts that mood fluctuations cause problems and wants to improve emotional balance and stability [stop rapid cycling, reduce impulsive behaviour, avoid severe manic or depressive episodes] Has and can use strategies to stabilise mood and emotions. |
| 6.05 | Increasing healthy behaviours and reducing unhealthy behaviours | A person recognises and develops patterns of behaviour that increase level of stability and wellness: valuing self: not blaming self; accepting personal worth and taking care of self, including appearance; finding enjoyment; taking relaxation and rewards. A person is able to recognise behaviour identified as personally unhealthy and likely to cause an episode of illness or crisis and take steps to reduce this. These behaviours are linked to their impact upon a person’s bipolar. |
| **7** | **MEDICATION EFFECTS** |  |
| 7.01 | Side-effects | The number, severity, impact of side-effects, including physical health, psychological and cognitive effects, including those which reduce sensitivity and make people feel blunted |
| 7.02 | Coping with side effects | A person understands the side effects of medications and is able to use strategies to reduce or mitigate them, including altering medication, in cooperation with health professionals |
| 7.03 | Satisfaction with medication* |  |
| **8** | **QUALITY OF LIFE** |  |
| 8.01 | Health-related quality of life | A state of health and well-being, which included physical, mental, emotional and social well-being. |
| 8.02 | Meaningful occupation and activities | A person is engaged in an occupation [paid or voluntary], training, education, or other activities which are meaningful to the individual and provide ongoing satisfaction and structure. |
| 8.03 | In control of finances | A person is in control of finances, including recognising the risk of, and taking steps to avoid, over-spending during a manic episode, paying bills on time, managing income, avoiding unmanageable debts and expenditure to meet own needs. |
| 8.04 | Personal safety and security | Being aware of personal impulsivity, vulnerability and risk factors associated with illness and has strategies to cope or to deal with them [e.g. seeking advice , deliberating] |
| 8.05 | Home living conditions and organisation | Having a home and living conditions that is sufficiently organised, clean and uncluttered to suit own personal needs |
| 8.06 | Vulnerability to harm | Risk or vulnerability to exploitation or harm from others. |
| **9** | **SERVICE OUTCOMES** |  |
| 9.01 | Relapse plans in place | A full a relapse plan is in place which has been agreed by the service user, professionals and carers. This describes what should happen if symptoms or ability to cope worsens, or a crisis occurs. |
| 9.02 | Timely and accurate diagnosis* |  |
| 9.03 | Number of days between referral and subsequent assessments* |  |
| **10** | **SERVICE USER EXPERIENCE OF CARE** |  |
| 10.01 | Dignity and respect | A person feels treated with dignity and respect by health and care professionals |
| 10.02 | Person’s overall satisfaction with service* |  |
| 10.03 | Shared decision making and control | A person feels in control of care, including negotiating treatment plans jointly with support from healthcare professionals. |
| 10.04 | Trusting patient & health professional relationship | Mutual trust in relationships between care professionals and service user |
| 10.05 | Actively involved with treatment and care plan | Is actively involved in own treatment and recovery, including making decisions, and attending appointments consistently. |
| **11** | **USE OF COERCION** |  |
| 11.01 | Use of coercion | Use of sectioning, restraint, coercion, isolation or seclusion to manage distress during hospital admission |

*These outcomes were not developed by the research group but were added by Delphi participants. Because of this, they do not have an accompanying definition developed by the research team and LEAP.
