# Supplementary material for: Development of a core outcome set for use in community-based bipolar trials—A qualitative study and modified Delphi
Source: PLoS One. 2020 Oct 28;15(10):e0240518. doi: 10.1371/journal.pone.0240518 (PMC7592842; doi:10.1371/journal.pone.0240518)
Supplement: S5 File — (DOCX) [file pone.0240518.s005.docx]

| **Stakeholder group** | **Delphi Round 1** | **Delphi Round 2** |
| --- | --- | --- |
| **People with bipolar diagnosis** | 15 | 12 |
| **Carers** | 4 | 3 |
| **Healthcare professionals** | 23 | 16 |
| **Researchers** | 8 | 7 |
| **Total** | 50 | 38 |
