# Supplementary material for: Development of a core outcome set for use in community-based bipolar trials—A qualitative study and modified Delphi
Source: PLoS One. 2020 Oct 28;15(10):e0240518. doi: 10.1371/journal.pone.0240518 (PMC7592842; doi:10.1371/journal.pone.0240518)
Supplement: S6 File — (DOCX) [file pone.0240518.s006.docx]

| **Stakeholder group** | **Gender** | **Ethnicity** | **Support from primary/secondary care for bipolar** | **Length of current mental health diagnosis** |
| --- | --- | --- | --- | --- |
| **People with bipolar diagnosis** | Male 2  Female 13 | White British 12  British Asian/Asian 1  Mixed Heritage 1  Not provided 1 | Primary 7  Secondary 7  Prefer not to say 1 | ≤2 years 1  3-10 years 8  11-20 years 3  21-30 years 1  31-40 years 2 |
| **Carers** | Male 1  Female 3 | White British 3  British Asian/Asian 1 | - | - |
| **Healthcare professionals** | Male 12  Female 11 | White British 19  British Asian/Asian 2  Not provided 2 | Primary 1  Secondary 2 | 11-20 years 2  Prefer not to say 1 |
| **Researchers** | Male 1  Female 7 | White British 8 | Primary 1 | 31-40 years 1 |
| **Total** | 50 | |  | |
