# Supplementary material for: Development of a core outcome set for use in community-based bipolar trials—A qualitative study and modified Delphi
Source: PLoS One. 2020 Oct 28;15(10):e0240518. doi: 10.1371/journal.pone.0240518 (PMC7592842; doi:10.1371/journal.pone.0240518)
Supplement: S7 File — (DOCX) [file pone.0240518.s007.docx]

| **Category** | **Domain** | **Proportion scoring 1-3 (%)** | **Proportion scoring 4-6 (%)** | **Proportion scoring 7-9 (%)** |
| --- | --- | --- | --- | --- |
| RECOVERY | Personal Recovery |  | 1 (3) | 37 (97) |
|  | Achieving goals |  | 2 (3) | 36 (97) |
|  | Sense of Identity |  | 7 (18) | 31 (82) |
|  | Hope |  | 2 (5) | 36 (95) |
|  | Meaning in Life |  | 7 (18) | 31 (82) |
|  | Empowerment |  | 7 (18) | 31 (82) |
|  | Wellbeing |  | 6 (16) | 32 (84) |
|  | Coping w Stigma |  | 12 (32) | 26 (68) |
|  | Coping w Self Stigma | 1 (3) | 6 (16) | 31 (82) |
|  | Self Esteem |  | 5 (13) | 33 (87) |
| CONNECTEDNESS | Trust |  | 8 (21) | 30 (79) |
|  | Relationships w F and F |  | 6 (16) | 32 (84) |
|  | Social Networks |  | 8 (21) | 30 (79) |
|  | Social Capital |  | 25 (66) | 13 (34) |
|  | Communication |  | 13 (34) | 25 (66) |
|  | Social Sensitivity | 2 (5) | 26 (68) | 10 (26) |
|  | Social Support |  | 5 (13) | 33 (87) |
|  | Social Isolation | 1 (3) | 6 (16) | 31 (82) |
|  | Loneliness |  | 11 (29) | 27 (71) |
| MENTAL HEALTH | Mental State |  | 5 (13) | 33 (87) |
|  | Mood Control and Stabilisation |  | 8 (21) | 30 (79) |
|  | Manic State |  | 6 (16) | 32 (84) |
|  | Paranoia |  | 9 (24) | 29 (76) |
|  | Anxiety | 1 (3) | 7 (18) | 30 (79) |
|  | Depression |  | 5 (13) | 33 (87) |
|  | Delusions |  | 9 (24) | 29 (76) |
|  | Unusual Behaviour | 1 (3) | 8 (21) | 29 (76) |
|  | Self-harm | 1 (3) | 5 (13) | 32 (84) |
|  | Psychological Pain and distress | 1 (3) |  | 37 (97) |
|  | Guilt and Shame | 1 (3) | 6 (16) | 31 (82) |
|  | Vulnerability to Harm |  | 3 (8) | 35 (92) |
|  | Relapse or Recovery Relapse |  | 3 (8) | 35 (92) |
| PHYSICAL HEALTH | All-cause Mortality | 3 (8) | 3 (8) | 32 (84) |
|  | Suicide |  | 1 (3) | 37 (97) |
|  | Mortality excluding suicide | 2 (5) | 5 (13) | 31 (82) |
|  | Physical Health |  | 6 (16) | 32 (84) |
|  | Physical fitness | 2 (5) | 16 (42) | 20 (53) |
| SELF MANAGEMENT | Self-management and understanding diagnosis |  | 1 (3) | 37 (97) |
|  | Increasing healthy behaviour |  | 3 (8) | 35 (92) |
|  | Reducing unhealthy behaviour | 1 (3) | 3 (8) | 34 (89) |
|  | Shared Decision-making and control | 2 (5) | 4 (11) | 32 (84) |
| MEDICATION | Self-management of medication |  | 7 (19) | 30 (81) |
|  | Medication Adherence | 1 (3) | 7 (19) | 29 (78) |
|  | Reduced use of medication | 4 (11) | 16 (43) | 17 (46) |
|  | Coping with side effects of medication |  | 4 (11) | 33 (89) |
|  | Side-effects |  | 6 (16) | 31 (84) |
|  | Weight control Side effect |  | 6 (16) | 31 (84) |
| QUALITY OF LIFE | Quality of life |  | 2 (5) | 35 (96) |
|  | Health Related QoL | 1 (3) | 5 (5) | 31 (84) |
|  | Able to build an everyday life |  | 1 (3) | 36 (97) |
|  | Meaningful occupation and activities |  | 3 (8) | 34 (92) |
|  | In control of finances |  | 8 (22) | 29 (78) |
|  | Personal Safety and security |  | 2 (5) | 35 (96) |
|  | Home living conditions and organisation |  | 8 (22) | 29 (78) |
|  | Hyper sexuality | 1 (3) | 11 (31) | 24 (66) |
| SERVICE OUTCOMES | Use of emergency care |  | 6 (17) | 30 (83) |
|  | Number of hospital bed use days | 2 (6) | 15 (42) | 19 (53) |
|  | Relapse |  | 4 (11) | 32 (89) |
|  | SU Experience of Care |  | 2 (6) | 34 (95) |
|  | Care plan in place | 3 (8) | 12 (33) | 21 (58) |
|  | Relapse plans in place | 1 (3) | 4 (11) | 31 (86) |
|  | Trusting Patient and HCP relationship |  | 2 (6) | 34 (95) |
|  | Dignity and Respect |  | 1 (3) | 35 (97) |
|  | Actively involved in t and care plan |  | 4 (11) | 32 (89) |
|  | Use of Coercion | 1 (3) | 9 (25) | 26 (72) |
|  | Measure of Use of All Services |  | 13 (36) | 23 (64) |
