# Supplementary material for: Development of a core outcome set for use in community-based bipolar trials—A qualitative study and modified Delphi
Source: PLoS One. 2020 Oct 28;15(10):e0240518. doi: 10.1371/journal.pone.0240518 (PMC7592842; doi:10.1371/journal.pone.0240518)
Supplement: S8 File — (DOCX) [file pone.0240518.s008.docx]

**Proposed outcomes:**

The table below contains four columns:

Column 1 (labelled: “Outcome previously listed in Delphi and rated as “important”) contains the outcomes that were rated as important by more than 70% of the Delphi participants during the Delphi. The outcomes that were not rated as important by more than 70% of the Delphi participants are NOT INCLUDED in this list because they did not make it through to this stage.

Column 2 (labelled: “Previous outcome listed in the Delphi”) shows how the outcomes in the Delphi were grouped into categories, or “domains”.

Column 3 (labelled: “Concepts covered when measuring suggested outcome”) contains the outcomes that were rated as important by more than 70% of the Delphi participants. However, some of the outcomes in this column have been merged by the research team and are now “concepts”. During the consensus meeting, you will be asked to think about whether you agree with how they have been merged.

Column 4 (labelled: “Suggested outcome”) contains the research team’s suggestion of how the concepts listed in Column 3 can be grouped together to form a broader “outcome”.

| COLUMN 1  Outcome previously listed in Delphi and rated as “important” | COLUMN 2  Domain previously used in Delphi |  | COLUMN 3  Concepts covered when measuring suggested outcome | COLUMN 4  Suggested outcome |
| --- | --- | --- | --- | --- |
| Personal Recovery | RECOVERY |  |  |  |
| Achieving goals |  |  | Achieving goals |  |
| Sense of Identity |  |  | Sense of Identity | Personal Recovery |
| Hope |  |  | Hope |  |
| Meaning in Life |  |  | Meaning in Life |  |
| Empowerment |  |  | Empowerment |  |
| Wellbeing |  |  | Wellbeing |  |
| Coping w Self Stigma |  |  | Coping w Self Stigma |  |
| Self Esteem |  |  | Self Esteem |  |
| Trust | CONNECTEDNESS |  | Trust |  |
| Relationships with friends and family |  |  | Relationships with friends and family |  |
| Social Networks |  |  |  | Social networks |
| Social Support |  |  | Social Support |  |
| Social Isolation |  |  | Social Isolation |  |
| Loneliness |  |  | Loneliness |  |
| Mental State | MENTAL HEALTH |  | Paranoia and delusions |  |
| Mood Control and Stabilisation |  |  | Anxiety and depression |  |
| Manic State |  |  | Manic state/unusual behaviour | Mental state/ psychological pain and distress |
| Paranoia |  |  | Mood control and stabilisation |  |
| Anxiety |  |  | Guilt and shame |  |
| Depression |  |  | Relapse or recovery response |  |
| Delusions |  |  |  |  |
| Unusual Behaviour |  |  |  |  |
| Self-harm |  |  |  |  |
| Psychological Pain and distress |  |  |  |  |
| Guilt and Shame |  |  |  |  |
| Vulnerability to Harm |  |  |  |  |
| Relapse or Recovery Relapse |  |  |  |  |
| All-cause Mortality | PHYSICAL HEALTH |  | Physical Health | Physical Health |
| Suicide |  |  | All-cause mortality | Mortality |
| Mortality excluding suicide |  |  | Self-harm | Adverse event |
| Physical Health |  |  | Suicide |  |
| Self-management and understanding diagnosis | SELF MANAGEMENT |  | Self-management and understanding diagnosis |  |
| Increasing healthy behaviour |  |  | Increasing healthy behaviour and reducing unhealthy behaviour |  |
| Reducing unhealthy behaviour |  |  | Shared decision-making and control | Self-management |
| Shared Decision-making and control |  |  | Self-management of medication and adherence  Trusting patient and HCP relationship  Actively involved in treatment and care-plan |  |
| Self-management of medication | MEDICATION |  | Side effects and coping with side-effects of medication |  |
| Medication Adherence |  |  | Weight control side effect |  |
| Coping with side effects of medication |  |  |  | Medication Side-effects |
| Side-effects |  |  |  |  |
| Weight control Side effect |  |  |  |  |
| Quality of life | QUALITY OF LIFE |  | Health Related Quality of Life |  |
| Health Related Quality of Life |  |  | Able to build an everyday life |  |
| Able to build an everyday life |  |  | Meaningful occupation and activities  In control of finances | Quality of life |
| Meaningful occupation and activities |  |  |  |  |
| In control of finances |  |  | Personal safety and security |  |
| Personal Safety and security |  |  | Vulnerability to harm |  |
| Home living conditions and organisation |  |  | Home living conditions and organisation |  |
| Use of emergency care | SERVICE OUTCOMES |  | Use of emergency care  Relapse plans in place  Timely and accurate diagnosis*  The number of days between referral and subsequent assessments* | Resource use |
| Relapse |  |  | Dignity and respect  Use of coercion  Person’s overall satisfaction with service* | Service user experience of care |
| SU Experience of Care |  |  |  |  |
| Relapse plans in place |  |  |  |  |
| Trusting Patient and HCP relationship |  |  |  |  |
| Dignity and Respect |  |  |  |  |
| Actively involved in t and care plan |  |  |  |  |
| Use of Coercion |  |  |  |  |

* These outcomes were added by Delphi participants in Round 1 and rated as important by the participants in Round 2.
