# Supplementary material for: Development of a core outcome set for use in community-based bipolar trials—A qualitative study and modified Delphi
Source: PLoS One. 2020 Oct 28;15(10):e0240518. doi: 10.1371/journal.pone.0240518 (PMC7592842; doi:10.1371/journal.pone.0240518)
Supplement: S9 File — (DOCX) [file pone.0240518.s009.docx]

***Outcome 1***

Suggested name: Personal recovery

Suggested arrangement:

- Achieving goals
- Sense of identity
- Hope
- Meaning in life
- Empowerment
- Wellbeing
- Self-esteem

| Round | Keep | Change | Discuss further |
| --- | --- | --- | --- |
| 1st | 50% | 36% | 14% |

New arrangement

New name: Personal recovery

- Achieving goals
- Sense of identity
- Hope
- Meaning in life
- Empowerment
- Coping with self-stigma
- Wellbeing
- Self-esteem*
- Able to build an everyday life

*Manuscript note required by meeting participants: This may overlap with “mental health” outcome

| Round | Keep | Change | Discuss further |
| --- | --- | --- | --- |
| 2nd | 93% | 7% | 0% |

***Outcome 2***

Suggested name: Social networks

Suggested arrangement:

- Trust
- Relationships with friends and family
- Social support
- Social isolation
- Loneliness

New arrangement

New name: Connectedness

- Satisfaction with social networks*
- Trust
- Relationships with friends, family and others
- Social support*
- Social isolation
- Loneliness*

*Manuscript note required by participants: Emphasise that despite being part of a social network, the person must be satisfied within theirs. Some participants were unhappy with the use of the term “repair” in the social network definition. Social support should be clarified so that this is a person’s social network rather than service provision (eg. Housing). Loneliness is a very important item that could even stand alone or could overlap with mental health.

| Round | Keep | Change | Discuss further |
| --- | --- | --- | --- |
| 1st | 79% | 0% | 21% |

First round vote prompted discussion about loneliness so a vote was held on whether it should be on its own:

| Round | Keep | Change | Discuss further |
| --- | --- | --- | --- |
| 2nd | 50% | 36% | 14% |

Decision: It was decided that loneliness should stay in Connectedness (as above) but should be emphasised as particularly important.

***Outcomes 3 and 4***

Suggested name: Mental state and psychological pain and distress

Suggested arrangement:

- Mood control and stabilisation
- Manic state and unusual behaviour
- Paranoia and delusions
- Anxiety and depression
- Guilt and shame
- Relapse or recovery response

New arrangement – split into two outcomes

New name: Clinical recovery of bipolar symptoms

- Paranoia
- Delusion
- Anxiety
- Depression
- Unusual behaviour
- Elevated mood*
- Relapse or recovery response

*Manuscript note required by participants: Elevated mood replaces manic state.

| Round | Keep | Change | Discuss further |
| --- | --- | --- | --- |
| 1st | 100% | 0% | 0% |

New name: Mental health and wellbeing

- Mental state
- Psychological distress
- Guilt and shame

| Round | Keep | Change | Discuss further |
| --- | --- | --- | --- |
| 1st | 79% | 7% | 14% |

Decision: Have two outcomes as above.

***Outcomes 5, 6, 7***

Suggested names: Mortality, Adverse Event, Physical Health

Suggested arrangement:

- All-cause mortality > “Mortality” outcome
- Self-harm, suicide > “Adverse event” outcome
- Physical health > “Physical health” outcome

New arrangement:

- All-cause mortality > “All-cause mortality” outcome
- Self-harm, attempted suicide, self-harm, use of emergency care > “Adverse event” outcome*
- Physical health* > “Physical health” outcome

*Manuscript note required by participants: Adverse events may not be classed as “outcomes” but as key safety indicators used by DMEC. Must add more detail, in particular the health concerns for people with bipolar including cardiovascular, metabolic, substance use etc. The focus of this will differ from trial to trial.

| Round | Keep | Change | Discuss further |
| --- | --- | --- | --- |
| 1st | 100% | 0% | 0% |

***Outcome 8***

Suggested name: Self-management

Suggested arrangement:

- Self-management and understanding diagnosis
- Increasing healthy behaviour and reducing unhealthy behaviour
- Shared decision-making and control
- Self-management of medication and adherence
- Trusting patient and healthcare professional relationship
- Actively involved in treatment and care plan

New arrangement

New name: Self-monitoring and management

- Self-management and understanding diagnosis
- Self-management of medication
- Medication adherence
- Mood control and stabilisation
- Increasing healthy behaviours and reducing unhealthy behaviours*

*Manuscript note required by participants: This requires rewording to better reflect definition and the link between behaviours and their impact on bipolar.

| Round | Keep | Change | Discuss further |
| --- | --- | --- | --- |
| 1st | 100% | 0% | 0% |

Decision: Later decided that adherence would be made its own item to indicate its importance.

***Outcome 9***

Suggested name: Medication side-effects

Suggested arrangement:

- Side-effects and coping with side-effects of medication
- Weight control side-effect

1^st^ vote:

Proposed name: Medication side-effects

- Side effects
- Coping with side-effects

| Round | Keep | Change | Discuss further |
| --- | --- | --- | --- |
| 1st | 57% | 36% | 7% |

2^nd^ vote:

Proposed name: Medication effects

- Side-effects
- Coping with side-effects
- Medication adherence*

*Manuscript note required by participants: Emphasis on satisfaction with medication and link with adherence

| Round | Keep | Change | Discuss further |
| --- | --- | --- | --- |
| 2nd | 93% | 7% | 0% |

This was not a true vote as 2 participants indicated they voted to “keep” despite not agreeing.

3^rd^ vote:

New name: Medication effects

- Side-effects
- Coping with side-effects
- Satisfaction with medication

| Round | Keep | Change | Discuss further |
| --- | --- | --- | --- |
| 3rd | 79% | 21% | 0% |

***Outcome 10***

Suggested name: Quality of life

Suggested arrangement:

- Health related quality of life
- Able to build an everyday life
- Meaningful occupation and activities
- In control of finances
- Personal safety and security
- Home living conditions and organisation
- Vulnerability to harm

New name: Quality of life

New arrangement:

- Health related quality of life
- Meaningful occupation and activities
- In control of finances
- Personal safety and security
- Home living conditions and organisation
- Vulnerability to harm

| Round | Keep | Change | Discuss further |
| --- | --- | --- | --- |
| 1st | 93% | 7% | 0% |

***Outcome 11, 12, 13, 14***

Suggested name: Resource use

- Use of emergency care
- Relapse plans in place
- Timely and accurate diagnosis**
- Number of days between referral and subsequent assessments**

Suggested name: Service user experience of care

- Dignity and respect
- Use of coercion
- Person’s overall satisfaction with service **

** Note: These were added in Round 1 of Delphi and rated as important in Round 2

New arrangement: These two outcomes should be split into four.

New name: Resource use

- To include all health service use including hospital admission, home treatment, outpatient use etc.

| Round | Keep | Change | Discuss further |
| --- | --- | --- | --- |
| 1st | 100% | 0% | 0% |

New name: Service outcomes

- Relapse plans in place
- Timely and accurate diagnosis
- Number of days between referral and subsequent assessments

| Round | Keep | Change | Discuss further |
| --- | --- | --- | --- |
| 1st | 86% | 7% | 7% |

New name: Service user experience of care

- Dignity and respect
- Person’s overall satisfaction with service
- Shared decision-making and control
- Trusting patient and healthcare professional relationship
- Actively involved in treatment and care plan

| Round | Keep | Change | Discuss further |
| --- | --- | --- | --- |
| 1st | 100% | 0% | 0% |

New name: Use of coercion

- Use of coercion

| Round | Keep | Change | Discuss further |
| --- | --- | --- | --- |
| 1st | 93% | 7% | 0% |
